# Host Status of Ornamental Shade Trees and Shrubs to Plant Parasitic Nematodes

**DOI:** 10.2478/jofnem-2024-0024

**Published:** 2024-06-28

**Authors:** T. Benedetti, J. E. Weiland, I. A. Zasada

**Affiliations:** Department of Horticulture, Oregon State University, Corvallis, OR 97330; USDA-ARS Horticultural Crops Disease and Pest Management Research Unit, Corvallis, OR 97331

**Keywords:** host-parasitic relationship, ornamental tree, shrubs, root-knot nematodes, *Meloidogyne incognita*, *Meloidogune hapla*, root lesion nematode, *Pratylenchus neglectus*

## Abstract

Oregon leads the United States in nursery production of shade trees and is third in deciduous and broadleaf evergreen shrub production. Plant-parasitic nematodes have been implicated in problems with the growth of plants in nurseries and are also of phytosanitary risk. A greenhouse experiment was conducted to evaluate the host status of four trees (*Quercus alba, Quercus garryana, Acer campestre, Thuja occidentalis*) and two shrubs *(Buxus sempervirens*, *Rhododendron catawbiense*) to *Meloidogyne incognita, Meloidogyne hapla*, and *Pratylenchus neglectus*. Each plant/nematode treatment was replicated five times, and the experiment was conducted twice. Plants were inoculated with 3,000 eggs of *M. incognita* or *M. hapla* and 2,500 individuals of *P. neglectus* two weeks after planting. After three months, the plants were harvested, and the total density of nematodes in soil and roots for *P. neglectus* and the total density of second-stage juveniles (J2) in soil and eggs on roots for *M. hapla* and *M. incognita* were determined. The final nematode population (Pf) and reproductive factor (RF = Pf/initial population density) were calculated. For *M. incognita* and *M. hapla*, all of the ornamental trees and shrubs would be considered as fair to good hosts with RF values > 1. *Meloidogyne incognita* had the highest Pf (5,234 total J2 and eggs/pot) and RF value (28.4) on *A. campestre*. For *P. neglectus*, all of the ornamental trees and shrubs were fair to good hosts, except for *B. sempervirens*. *Buxus sermpervirens* was not a host for *P. neglectus,* with an RF value of almost 0. This is the first report of *Q. alba, Q. garryana*, and *A. campestre* as hosts for *M. incognita, M. hapla*, and *P. penetrans*. This is also the first report of *T. occidentalis* and *R. catawbiense* as hosts for *P. penetrans* and the non-host status of *B. sermpervirens* for *P. penetrans*.

There is much information on the plant–parasitic nematodes associated with and impacting agricultural crops. However, the occurrence and impact of plant-parasitic nematodes on ornamental trees and shrubs have not been well defined. Considering the growth of the ornamental industry and the increasing demand for ornamental plants, this is an important knowledge gap (Howland and Quintanilla, 2023). The ornamental plant industry is valued at $70 billion worldwide (Madhavan et al., 2021). The United States is one of the leading ornamental producers in the world. Oregon leads the United States in nursery production of shade trees ($118 million) and is third in broadleaf evergreen shrubs ($91 million) (USDA NASS 2020). *Acer* spp. (maple) and *Quercus* spp. (oak) production alone is valued at $194 million and $184 million, respectively, and are the most valuable tree species produced nationwide. *Buxus sempervirens* (boxwood) is the most valuable broadleaf evergreen ($141 million), while *Thuja occidentalis* (arborvitae) is the most valuable ornamental conifer ($155 million).

This study focused on five important ornamental shrubs and trees produced in the U.S.: *Quercus alba* (white oak), and *Q. garryana* (Oregon oak), *Acer campestre* (hedge maple), *B. sempervirens, Rhododendron catawbiense* (rhododendron), and *T. occidentalis*. The amount of information that is available on the host status of these species to plant-parasitic nematodes varies. No information is available on the nematode parasites associated with *Q. alba* and *Q. garryana*. Only limited information exists on plant-parasitic nematodes associated with *A. campestris*, with reports of this species being susceptible to *Meloidogyne chitwoodi*, *Pratylenchus penetrans,* and two species of *Xiphinema* (Goodey et al., 1965; Den Nijs et al., 2004; Arias et al., 2005; Barsi and De Luca, 2008). There have been many plant-parasitic nematodes reported to be associated with *B. sempervirens*, *T. occidentalis*, and *R. catawbiense*, including *Meloidogyne* spp., *Pratylenchus* spp., *Tylenchorynchus* spp., *Paratrichodorus* spp., *Mesocriconema* spp., and *Xiphinema* spp. (Goodey et al., 1965; Siddiqui et al., 1973; Benson and Barker, 1982; Bernard and Witte, 1987; Den Nijs et al., 2004).

The goal of this research was to further define the host status of important ornamental trees and shrubs to plant-parasitic nematodes. We focused on three plant-parasitic nematode species that have the potential to be found in nursery production fields: *Meloidogyne hapla*, *Meloidogyne incognita*, and *Pratylenchus neglectus*.

## Materials and Methods

*Meloidogyne incognita* used in this trial was originally isolated from a grape vineyard in Parlier, CA, and *M. hapla* was originally isolated from a grape vineyard in Mattawa, WA. Both populations were obtained from single-egg masses and maintained on tomato (*Solanum lycopersicon* ‘Rutgers’). Species were confirmed by North Carolina Department of Agriculture and Consumer Services (Raleigh, NC) using molecular methods. Inoculum was obtained by destructively harvesting tomato plants and collecting eggs from washed roots by shaking roots in 0.05% NaOCl solution for 3 min. The egg solution was then poured over nested 250- and 25-μm sieves, with eggs retained on the 25-μm sieve. Eggs were collected in water and adjusted to achieve desired inoculation densities. *Pratylenchus neglectus* was originally collected from a wheat field in Pendleton, OR, and the population was derived from a single individual and identified to species (Yan et al., 2013). The culture was maintained on carrot discs as previously described (Moody et al., 1973). To extract the nematodes from the carrot discs, the discs were cut into small pieces, placed into a blender, covered with water, and blended. The slurry was then placed on a 25-μm sieve over water, and nematodes extracted for 12 to 48 hours. Collected *P. neglectus* were concentrated on a 28-μm sieve and adjusted to achieve the desired inoculation density.

Trees and shrubs were obtained as dormant, young plants from commercial nurseries in February and March 2023. *Quercus alba, Q. garryana*, and *A. campestre* were obtained as 15-cm tall, 1-year-old seedlings. *Thuja occidentalis* ‘Green Emerald’, *B. sempervirens* ‘Green Velvet’, and R. *catawbiense* ‘Boursault’ were obtained as 15–30 cm tall, 1- to 2-year-old rooted cuttings. Prior to planting, potting media was rinsed from roots, and then the plants were transplanted into 3-L pots containing a pasteurized 1:1 sand to Willamette loam mix.

Two experiments were conducted with each plant/nematode combination replicated five times; experiments were separated by time of inoculation but maintained in the same greenhouse. Plants were grown in a greenhouse with a 16 h: 8 h, light/dark photoperiod. Temperature in the greenhouse was set to 25°C during the day and 20°C at night. Plants were fertigated biweekly with a water-soluble fertilizer (20N-20P-20K delivering 200 ppm N; Jack's, Allentown, PA) for the duration of the experiment. Two weeks after transplanting, the plants were inoculated with plant-parasitic nematodes. The initial inoculation density for *M. hapla* and *M. incognita* was 3,000 eggs/pot, and for *P. neglectus* was 2,500 nematodes (mixed life stages)/pot. The inoculum was placed close to the root system of the plants in several holes at a depth of approximately 2.5 cm. Tomato ‘Rutgers’ was used as a positive control for *Meloidogyne* spp., and wheat ‘Scarlet’ was used as a positive control for *P. neglectus*. Pots were blocked by nematode species and then arranged in a completely randomized design.

After three months, the aboveground portion of the plants was removed, and the roots were rinsed free of soil under running tap water. *Meloidogyne* spp. eggs were extracted from roots by a modified bleach extraction method (Hussey and Barker, 1973). Approximately 10 g of roots were placed in a container, covered with a 10% NaOCl solution, and shaken for 3 min. The resulting egg suspension was poured through 75- and 25-μm sieves with eggs retained on the latter sieve. Eggs were rinsed into a tube. To extract *P. neglectus*, 10 g of roots were placed under intermittent mist (15 sec mist every 2 min) for five days (Zasada et al., 2015). All extracted nematodes were stored at 4ºC until counted. The remainder of the root system was placed in a 70ºC oven for three days and then weighed; the 10-g root subsamples used for extractions were treated the same. The root weights were combined to determine the dry weight of the entire root system. Second-stage juveniles (J2; for *Meloidogyne* spp.) or mixed-stage individuals (for *P. neglectus*) were extracted from soil with the Baermann-funnel method (Baermann, 1917) by placing 50 g of soil on a funnel for 5 days.

To obtain the *M. incognita* and *M. hapla* final nematode densities (Pf), the total number of J2 in the soil was extrapolated from the number of J2 extracted in 50 g of soil, and the total number of eggs in the entire root system was extrapolated from the number of eggs in 10 g of roots. To obtain the *P. neglectus* Pf, the total number of nematodes in the soil was extrapolated from the number of *P. neglectus* extracted in 50 g of soil, and the total mixed stages of *P. neglectus* in the entire root system was extrapolated from the number of mixed stages of *P. neglectus* extracted from 10 g of roots. The host efficiency was determined by the reproduction factor (RF) = Pf/Pi, which was calculated where Pf = final nematode population density and Pi = the initial nematode population density. A reproduction factor greater than one indicated an increase in nematode reproduction, whereas an RF factor of less than one indicated no increase in reproduction. Host suitability was categorized as good [susceptible] when Pf/Pi > 5.0, fair [moderately susceptible] if 5.0 ≥ Pf/Pi > 1, poor [moderately resistant] if 1 > Pf/Pi > 0, and non-host [resistant] when Pf/Pi = 0 (Zhang and Schmitt, 1994).

Data from the two trials was combined for analysis. Data homogeneity was assessed by the Kolmogorov–Smirnov test, and normality was assessed by the Bartlett test. The data was analyzed by the Kruskal-Wallis test followed by the post hoc test of Duncan's multiple range test (*P* ≤ 0.05). The statistical analyses were performed using R Studio software (R Studio Team, 2021).

## Results

Across all of the plant-parasitic nematode species considered, the positive control plants (tomato for *Meloidogyne* spp. and wheat for *P. neglectus*) were significantly better hosts than any of the ornamental trees or shrubs (Tables 1, 2, and 3). Final nematode densities on the controls were > 44-fold higher than on the ornamental trees and shrubs, indicating that the control plants were excellent hosts for the nematodes considered in this study.

**Table 1.** *Meloidogyne incognita*, *M. hapla*, and *Pratylenchus penetrans* densities in soil and roots of ornamental tree and shrub.

**Host plant**	**Number of nematodes recovered from soil**			**Number of eggs recovered from roots**		**Number of nematodes recovered from roots**
		
	***M. incognita***	***M. hapla***	***P. neglectus***	***M. incognita***	***M. hapla***	***P. neglectus***
*Acer campestre*	35,640 ± 425 b^a^	17 ± 371 a	8,360 ± 222 b	49,595 ± 869 bc	5628 ± 122 a	4,050 ± 373 c
*Thuja occidentalis*	11,528 ± 141 a	30 ± 38 a	20,592 ± 627 bc	485 ± 109 a	6,409 ± 70 a	113 ± 15 ab
*Buxus sempervirens*	13,904 ± 119 ab	11 ± 222 a	0 ± 0 a	3,094 ± 354 b	1,935 ± 37 a	81 ± 21 a
*Quercus alba*	59,576 ± 868 b	24 ± 383 a	15,004 ± 360 bc	132 ± 14 a	5,938 ± 98 a	821 ± 126 b
*Quercus garryana*	30,008 ± 362,3 ab	24 ± 383 a	9,856 ± 312 bc	108 ± 14 a	1,660 ± 22 a	80 ± 13 a
*Rhododendron catawbiense*	23,584 ± 213 ab	13 ± 150 a	3,344 ± 450 b	3,864 ± 334 b	1,311 ± 16 a	71 ± 12 d
*Control* ^b^	799,128 ± 8,126 c	561 ± 4,689 b	149,547 ± 18,878 d	32,048 ± 319 c	77,391 ± 659 b	445,350 ± 25,049 d

a Data are presented as a mean ± standard error of 10 replications. Data were analyzed with the Kruskal-Wallis test with a post hoc test of Duncan at a 5% significance level. Values followed by different letters within a column indicate a statistical difference.

b Tomato for *Meloidogyne* spp. and wheat for *P. neglectus*.

**Table 2.** Final *Meloidogyne incognita*, *M. hapla*, and *Pratylenchus penetrans* densities and reproductive factor (RF)^a^ values in ornamental trees and shrubs.

**Host plant**	**Final population density**			**RF^a^**		
	
	***M. incognita***	***M. hapla***	***P. neglectus***	***M. incognita***	***M. hapla***	***P. neglectus***
*Acer campestre*	85,235 ± 118 b^b^	22,524 ± 492 a	12,410 ± 214 c	28 ± 4 b	8 ± 2 a	5 ± 9 cd
*Thuja occidentalis*	12,013 ± 104 a	36,945 ± 485 a	20,705 ± 627 ab	4 ± 5 a	12 ± 16 a	8 ± 3 d
*Buxus sempervirens*	16,998 ± 105 ab	13,375 ± 259 a	81 ± 211 a	6 ± 4 ab	5 ± 9 a	0 ± 0 a
*Quercus alba*	59,708 ± 869 b	30,226 ± 480 a	15,825 ± 357 bc	20 ± 3 b	10 ± 16 a	6 ± 1 cd
*Quercus garryana*	30,116 ± 362 ab	11,614 ± 175 a	9,926 ± 311 ab	10 ± 1 ab	4 ± 6 a	4 ± 1 bcd
*Rhododendron catawbiense*	27,448 ± 231 ab	14,335 ± 166 a	3,415 ± 45 b	10 ± 8 ab	5 ± 6 a	1 ± 18 bc
Control^c^	831,176 ± 8,044 c	638,805 ± 5,291 b	594,897 ± 3,696 d	277 ± 27 c	213 ± 176 b	238 ± 148 e

a RF = final population density/initial population density.

b Data are presented as a mean + standard error of 10 replications. Data were analyzed with the Kruskal-Wallis test with a post hoc test of Duncan at a 5% significance level. Values followed by different letters within a column indicate a statistical difference.

c Tomato for *Meloidogyne* spp. and wheat for *P. neglectus*.

**Table 3.** Host suitability of trees and shrubs seedlings for *Meloidogyne incognita*, *M. hapla*, and *Pratylenchus penetrans*.

**Host plant**	**Host suitability^a^**		

	***M. incognita***	***M. hapla***	***P. neglectus***
*Acer campestre*	S	S	MS
*Thuja occidentalis*	MS	S	S
*Buxus sempervirens*	S	MS	R
*Quercus alba*	S	S	S
*Quercus garryana*	S	MS	MS
*Rhododendron catawbiense*	S	MS	MS
Control^b^	S	S	S

a Host suitability was categorized as good [susceptible; S] when final population density (Pf)/initial population density (Pi) > 5.0, fair [moderately susceptible; MS] if 5.0 ≥ Pf/Pi > 1, poor [moderately resistant; MR] if 1 > Pf/Pi > 0, and non-host [resistant; R] when Pf/Pi = 0 (Zhang and Schmitt, 1994).

b Tomato for *Meloidogyne* spp. and wheat for *P. neglectus*.

**Figure 1.**
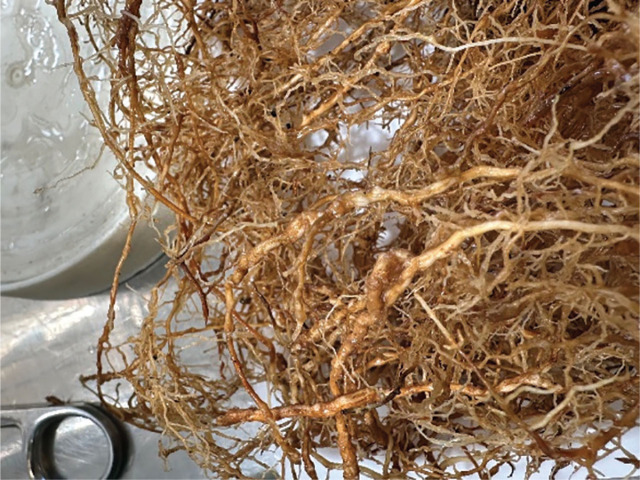
*Meloidogyne incognita* galls on *Acer campestre* roots.

All the ornamental trees and shrubs supported the reproduction of *M*. *incognita* (Table 1). The number of *M. incognita* J2 recovered from soil was relatively consistent across trees and shrubs. However, final egg population densities recovered from roots varied among plants. Significantly fewer eggs were recovered from *Q. alba, Q. garryana,* and *T. occidentalis* compared to *A. campestre*, *B. sempervirens,* and *R. catawbiense* (Table 1). *Acer campestre* was the plant species with evident symptoms of *M. incognita* parasitism in the root system (Fig. 1). When soil and root population densities of *M. incognita* were combined, *T. occidentalis* was a poorer host for *M. incognita* compared to *A. campestre* and *Q. alba* based upon Pf and RF values (*P* < 0.05). The remaining ornamental trees and shrubs were intermediate in host status. There was no variation in densities of *M. hapla* recovered from soil (J2) or roots (eggs) among the ornamental tree and shrub species (Table 1). Based on Pf and RF values, all of the ornamental trees and shrubs were similar in their ability to host *M. hapla* (Table 2).

The most variation in host status of the ornamental trees and shrubs was observed for *P. neglectus*. Similar densities of *P. neglectus* were recovered from soil for all of the trees and shrubs except for B. sempervirens where no nematodes were recovered (Table 1). Similarly, fewer *P. neglectus* were recovered from *B. sempervirens* roots, but at similar densities as for *R. catawbiense*, *Q. garryana*, and *T. occidentalis*. *Acer campestre* roots supported the highest densities of *P. neglectus*, at a level greater than 50 times the other ornamental trees and shrubs. Total Pf and RF values showed that *B. sempervirens* supported low *P. neglectus* densities, with an RF value significantly different than all of the other trees and shrubs (*P* < 0.05; Table 2).

Combined, the data allowed us to assign a susceptibility/host status designation to the ornamental trees and shrubs for all of the plant-parasitic nematodes (Table 3; Zhang and Schmitt, 1994). Only *B. sempervirens* was considered resistant to any of the nematodes, with almost no *P. neglectus* recovered from the plants at the end of the experiment.

## Discussion

This experiment evaluated the host status of some of the leading ornamental shade trees and shrubs grown in the United States to plant-parasitic nematodes. All of the woody perennial trees and shrubs evaluated here would be considered hosts for *M. incognita* and *M. hapla*, with RF values > 1; however, the degree of host status varied among the plants considered. The host status of the woody ornamentals to *P. neglectus* was more variable. *Buxus sempervirens* was not a host for *P. neglectus*, with the other woody ornamentals ranging from poor to good hosts for the nematode. To our knowledge, the host status of the two oaks considered in this study, *Q. alba* and *Q. garryana*, for plant-parasitic nematodes has not previously been considered. This is the first report of these two oak species as hosts for *M. incognita, M. hapla,* and *P. neglectus*. Only a limited amount of information is available on the host status of *A. campestre* to plant-parasitic nematodes, and this is the first report of this maple species as a host for *M. incognita, M. hapla,* and *P. neglectus. Buxus sempervirens* has already been reported as a host for *M. incognita* and *M. hapla,* but the nonhost status of boxwood for *P. neglectus* is new information. The susceptible host status of *T. occidentalis* and *R. catawbiense* for *P. neglectus* is also new information.

Five of six tree and shrub species evaluated in this trial were susceptible to and good hosts for *M. incognita*. Four plant species (*A. campestre, B. sempervirens, Q. garryana*, and *R. catawbiense*) had higher RF values for *M. incognita* than for *M. hapla* and *P. neglectus*. These results confirm the broad host range and aggressive nature of *M. incognita* in ornamentals (Anwar and McKenry, 2010; Muhae-ud-Din et al., 2018). The mortality of trees in plantations with high population densities of *M. incognita* has been reported (Wang et al., 1975; Saucet et al., 2016; Khan, 2020; Tanimola and Ezeunara, 2021). The same susceptible host status was observed for *M. incognita* for two species of oak (*Q. alba* and *Q. garryana*), *B. sempervirens*, and *R. catawbiense*. It is interesting that both oak species supported higher *M. incognita* J2 densities in the soil but, in contrast, had lower egg densities in the root system. The expected correlation between the number of *M. incognita* J2 and females in the root did not occur. The timing of sampling may have affected nematode development. High *M. incognita* J2 densities may indicate that the females had died (Gabia et al., 2015). Previous literature reports *M. incognita* parasitizing *Acer* spp. (Riffle, 1963; Powell, 1971; Muhammad and Khan, 2022), *Quercus* spp. (Santamour, 1992; Chalanska and Labanowski, 2014), and *B. sempervirens* (Siddiqui et al., 1973; Bernard, 1980; Sharma and Rich, 2005; Brito et al., 2010; Eisenback, 2018). In our study, *T. occidentalis* was moderately susceptible to *M. incognita* and a poorer host for the nematode than other trees and shrubs. There were large differences in the frequency of nematode established within all gymnosperm and angiosperm families. In general, conifers are poor hosts for plant-parasitic nematodes, while there is a larger percentage of plant-parasitic nematodes that parasitize angiosperms (Eschen et al., 2015). Among the angiosperms, high population densities of plant-parasitic nematodes have been reported on *Aceraceae*, *Fagaceae*, and *Rutaceae*. *Meloidogyne incognita* also prefers to parasitize herbaceous dicotyledon species rather than lignified ones (Rathore and Ali, 2014).

*Meloidogyne hapla* is widely distributed, particularly in temperate regions and the cooler, higher-altitude areas of the tropics (Goodey et al., 1965). In the U.S., *M. hapla* is reported to infect over 550 crops and weeds (Taylor and Buhrer, 1958). *Acer campestre*, *T. occidentalis*, and *Q. alba* were all susceptible and good hosts for *M. hapla*. These plants have already been reported as hosts for *M. hapla* (Riffle, 1963; Powell, 1971; Sohrabi et al., 2015; Muhammad and Khan, 2022). *Quercus garryana*, *B. sempervirens*, and *R. catawbiense* were moderately susceptible to *M. hapla.* The results for *Q. garryana* and *B. sempervirens* align with the literature that reported these species were hosts for *M. hapla* (Bernard and Witte, 1987). However, our determination that *R. catawbiense* ‘Boursault’ is a host for *M. hapla* contradicts the findings of Bernard and Witte (1987) where the same variety was immune and not a host for *M. hapla*. This difference may be due to differences in the pathogenicity of the *M. hapla* populations used in the studies.

*Pratylenchus* spp. are frequently found in soil from woody ornamentals, often in high densities and associated with plant decline (Bernard, 1980). However, *Pratylenchus* sp. densities associated with plants normally decreased as trees matured (Manlay et al., 2000). Our data showed that *Q. garryana* and *R. catawbiense* were moderately susceptible to *P. neglectus*. This suggests that the nematode was able to penetrate the root, but some root factor reduced their development (Vicente and Acosta, 1987). *Pratylenchus neglectus* has been demonstrated to be harmful to *Acer* spp. production (Chalanska and Labanowski, 2014). Dieback of *Rhododendron* sp. was attributed to nematode parasites, including *Pratylenchus* (Baird et al., 2014). *Quercus alba* and *T. occidentalis* were susceptible to *P. neglectus,* although the literature reports that the *Pratylenchus* spp. are potentially more harmful to *T. occidentalis* than were *P. crenatus*, *P. projectus*, and *P. nanus* (Chalanska and Labanowski, 2014). *Quercus* spp. have also been reported as a host for *P. neglectus,* but not specifically the species considered here (Siddiqui et al., 1973; Chalanska and Labanowski, 2014; Mehrabian et al., 2020). *Buxus sempervirens* was resistant to *P. neglectus*. This result contradicts literature that reported *Pratylenchus* spp. parasitizing *B. sempervirens* (Taylor, 1944; Benson and Barker, 1982; Lehman, 1984; Lopez-Nicora et al., 2012; Eisenback, 2018). In these studies, *B. sempervirens* was a host for *P. vulnus, P. penetrans, P. pratensis,* and *P. coffeae* (Benson and Barker, 1982; Goodey et al., 1965). Differences in parasitism among the species may be attributed to genetic diversity among the different *Pratylenchus* spp. or genetic differences between the host species (Brown et al., 1980; Kayani and Mukhtar, 2018; Azizi, 2022).

Other *Meloidogyne* and *Pratylenchus* spp. have been reported to parasitize the ornamental trees and shrubs considered in this study. *Acer campestre* was a host for *M. chitwoodi* (Den Nijs et al., 2004). *Rhododendron* spp. were susceptible hosts for *P. vulnus*, *P. crenatus*, and *M. pini* (Eisenback et al., 1985; Siddiqui et al., 1973). *Thuja occidentalis* has already been reported to be parasitized by *P. penetrans* and *M. incognita* (Goodey, 1965; Wang et al., 1975). *Quercus* spp. was described as a host for *M. partytula* (Eisenback et al., 2015), and *B. sempervirens* is susceptible to many nematodes, including *M. arenaria*, *P. penetrans*, *P. vulnus*, *P. pratensis*, *P. coffee*, *M. thanesi*, *M. incognita*, *M. fallax*, and *M. chitwoodi* (Siddiqui et al., 1973; Benson, 1985; Den Nijs et al., 2004).

This study focused on three plant-parasitic nematodes that impact the ornamental plant industry, but there are many other plant-parasitic nematodes with unknown economic and damage potential in this field. Further research on infection behavior, overwintering survival, and nematode epidemiology is needed to better manage nematodes and meet the growing demand for ornamental plants. By controlling these nematodes, we can prevent their spread through exports and minimize global yield loss.
